# Single isocenter stereotactic irradiation for multiple brain metastases: current situation and prospects

**DOI:** 10.1007/s11604-022-01333-7

**Published:** 2022-09-03

**Authors:** Megumi Uto, Daichi Torizuka, Takashi Mizowaki

**Affiliations:** Department of Radiation Oncology and Image-Applied Therapy, Kyoto University Graduate School of Medicine, 54, Shogoin Kawahara-cho, Sakyo-ku, Kyoto, 606-8507 Japan

**Keywords:** Single isocenter stereotactic irradiation, Multiple brain metastases, Linear accelerators

## Abstract

The prognosis of patients with brain metastases has dramatically improved, and long-term tumor control and reduction of the risk of late toxicities, including neurocognitive dysfunction, are important for patient quality of life. Stereotactic irradiation for multiple brain metastases, rather than whole-brain radiotherapy, can result in high local control rate with low incidence of neurocognitive deterioration and leukoencephalopathy. Recent advances in radiotherapy devices, treatment-planning systems, and image-guided radiotherapy can realize single isocenter stereotactic irradiation for multiple brain metastases (SI-STI-MBM), in which only one isocenter is sufficient to treat multiple brain metastases simultaneously. SI-STI-MBM has expanded the indications for linear accelerator-based stereotactic irradiation and considerably reduced patient burden. This review summarizes the background, methods, clinical outcomes, and specific consideration points of SI-STI-MBM. In addition, the prospects of SI-STI-MBM are addressed.

## Introduction

Brain metastases are common in cancer patients, with an incidence of 9.6% of all cancer patients [1]. Brain metastases and peritumor edema often cause severe neurological symptoms, which directly affect the patients’ quality of life. Surgery, systemic therapy, radiotherapy, and the best supportive care are the treatment options for patients with brain metastases. Although surgery can be a treatment option for brain metastases generally larger than 3 cm in diameter, complications due to surgery are sometimes severe, and the risks of decline in performance status and neurological function should be considered. In contrast, radiotherapy has been used as a useful and less invasive local therapy for brain metastases [2].

Whole-brain radiotherapy (WBRT) used to be the sole standard radiotherapeutic approach for patients with brain metastases, and the intent and irradiation dose of WBRT are palliative rather than curative. This is because the whole brain is irradiated during WBRT, and ablative doses to the whole brain inevitably result in severe acute and late toxicities. Despite only palliative irradiation doses, neurocognitive dysfunction and leukoencephalopathy due to WBRT should be considered, because these late complications are sometimes observed and directly affect patient’s lives [3]. Recently, prognosis of patients with brain metastases has dramatically improved owing to advances in systemic therapy [4]. Therefore, long-term brain tumor control and reduction of the risks of late complications caused by radiotherapy are important for improving patient quality of life. Thus, ablative local radiotherapy for brain metastases has become increasingly important and is used in clinical practice.

Stereotactic irradiation was developed and clinically used as an alternative ablative radiotherapy option for intracranial lesions using the Leksell Gamma Knife System for gamma knife radiosurgery (GKS) [5]. In addition to the gamma knife, linear accelerators (LINAC) can deliver stereotactic irradiation for brain metastases using circular cones and multileaf collimators [6, 7]. In GKS- and conventional LINAC-based stereotactic irradiation, tumors are irradiated lesion-by-lesion, the number of brain metastases increases, and the total treatment time increases. Recently, owing to advances in image-guided radiotherapy and treatment-planning devices, LINAC-based stereotactic irradiation can deliver a precise dose distribution with excellent conformity and steep dose gradient in a shorter time and in a less invasive manner [8]. Only 1–4 brain metastases have been treated with LINAC so far, because of technical challenges and prolonged treatment time. However, sophisticated optimization in treatment-planning systems has realized stereotactic irradiation for multiple brain metastases with only one isocenter: single isocenter stereotactic irradiation for multiple brain metastases (SI-STI-MBM) [9]. Thus, more than four brain metastases become technically treatable using SI-STI-MBM. Here, the current situation and prospects of SI-STI-MBM are reviewed.

## Improvement of prognosis of patients with brain metastases

Prognosis of patients with brain metastases was limited before 2000s. In 1997, Gasper et al. reported the recursive partitioning analysis (RPA) classification, which showed the prognosis of patients with brain metastases treated with radiotherapy [10]. The survival rate at 20 months was close to 15% even in the best prognosis group (RPA class 1).

Thereafter, other prognostic classifications have been published: GPA [11], disease-specific GPA [12], lung-mol GPA [4], updated Lung-GPA [13], melanoma mol-GPA [14], GI-GPA [15], and breast GPA [16]. Lung-mol GPA was advocated in 2017, 20 years after publication of the RPA classification. In the lung-mol GPA group, the survival rate at 20 months was similar between the worst group (GPA 3.5–4) and RPA class 1. In contrast, the best group (GPA 0–1) showed a survival rate close to 80% at 20 months, and the median overall survival was 46.8 months.

To improve the prognosis of patients with brain metastases, clinicians should pay more attention to late toxicities caused by radiotherapy. Neurocognitive dysfunction is a major and serious late complication of WBRT [17]. In contrast, stereotactic irradiation has little impact on neurocognitive dysfunction when compared with WBRT and can deliver an ablative irradiation dose to the tumor, which leads to long-term local control. Therefore, stereotactic irradiation is used more often than in the past, especially in patients with a good prognosis and a few lesions. From the point of view of the technical aspects of LINAC and clinical evidence on stereotactic irradiation in multiple brain metastases, up to four brain metastases are candidates for LINAC-based stereotactic irradiation.

## Stereotactic irradiation for up to ten brain metastases

In several randomized control trials involving patients with WBRT and stereotactic irradiation, the overall survival in patients with 1–4 brain metastases was similar regardless of whether the patients received WBRT and/or stereotactic irradiation [18–22]. Although 1–4 brain metastases are commonly treated with stereotactic irradiation for long-term tumor control and reduction of late toxicities, patients with four or more brain metastases are only treated with gamma knife radiosurgery in a limited number of institutions [21, 23, 24]. However, Yamamoto et al. reported a multi-institutional prospective observational study, termed JLGK0901, in which patients with up to ten brain metastases were treated with stereotactic radiosurgery (SRS) alone [25]. That study investigated GKS in patients with 1–10 brain metastases and showed a similar overall survival between patients with 2–4 brain metastases and patients with 5–10 brain metastases. The study included patients with the largest tumor with a volume of 10 cm^3^ or less and a longest diameter of 3.0 cm or less. In addition, the cumulative volume of all tumors was limited to 15.0 cm^3^ or less. Based on the JLGK0901 study, the NCCN guidelines developed the concepts of “limited brain metastases” and “extensive brain metastases” in 2018. “Limited brain metastases” is defined as a group of patients for whom SRS is equally effective and offers significant cognitive protection when compared with WBRT. SRS is preferred over WBRT in patients with limited brain metastases. Thus, up to ten brain metastases are now often treated with stereotactic irradiation.

## Development of SI-STI-MBM for multiple brain metastases

In LINAC-based stereotactic irradiation, brain metastases are conventionally treated with conformal arcs, one by one [6]. Accordingly, treatment time increases as the number of brain metastases increases. Considering the increased treatment time and burden in patients with frame-based fixation or oppressive frameless thermoplastic masks, only to 1–4 brain metastases could be treated with LINAC-based stereotactic irradiation.

Owing to advances in image-guided radiotherapy and optimization methods for treatment-planning systems, SI-STI-MBM can be performed in clinical settings [26]. In SI-STI-MBM, only one isocenter was sufficient, and multiple brain metastases were irradiated simultaneously. Therefore, the treatment time was dramatically reduced in SI-STI-MBM when compared with GKS and dynamic conformal arc therapy lesion-by-lesion [26–29]. By shortening the treatment time, the burden on patients can be reduced.

**Fig. 1:**
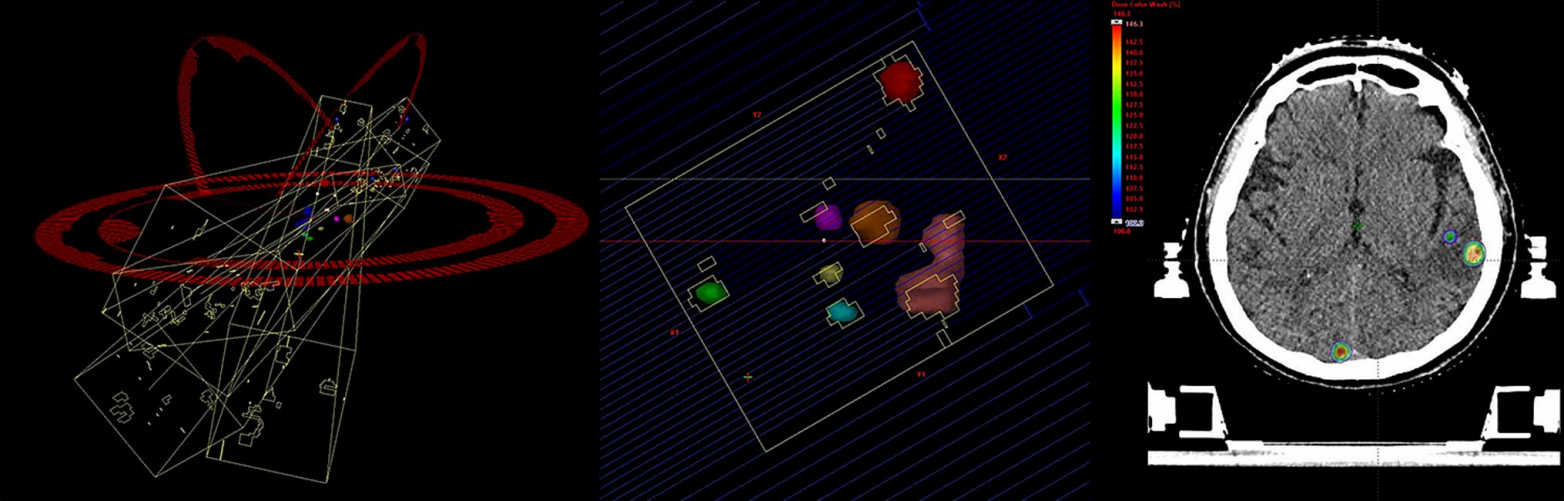
Single isocenter stereotactic irradiation using VMAT

Currently, two irradiation approaches are clinically used for SI-STI-MBM. The first is the volumetric-modulated arc therapy (VMAT) (Fig. 1). By using VMAT for SI-STI-MBM, dose distribution for multiple brain metastases with better conformity and steep dose fall-off can be made, even if tumors have irregular shapes or large maximum diameters [30]. If two brain metastases are close to each other, it is challenging to reduce the dose delivered to the normal brain between the two lesions. However, VMAT can decrease the irradiated dose of normal tissue as much as possible while maintaining conformity and a steep gradient using dose-intensity modulation and inverse-planning methods. Although VMAT plans can be manually generated by modifying the optimization objects for each lesion and index, knowledge-based planning (KBP) can help clinicians to generate SI-STI-MBM with VMAT plans [31]. In SI-STI-MBM with VMAT plans, inverse-planning methods and dose-intensity modulation can realize that all multiple targets are covered by the prescribed dose while maintaining high conformity, steep dose fall-off, and a high maximum dose for each site. However, making SI-STI-MBM plans places a large burden on radiation oncologists and medical physicists and may limit the chances of timely treatment initiation in patients with multiple brain metastases. RapidPlan™ (Varian Medical Systems, Palo Alto, CA, USA) is a KBP product that utilizes a machine learning system to establish a model to predict dose-volume histograms. RapidPlan™ enables less-experienced physicians to easily create high-quality plans in a short time [31, 32]. RapidPlan™ can be used to make SI-STI-MBM plans.

**Fig. 2:**
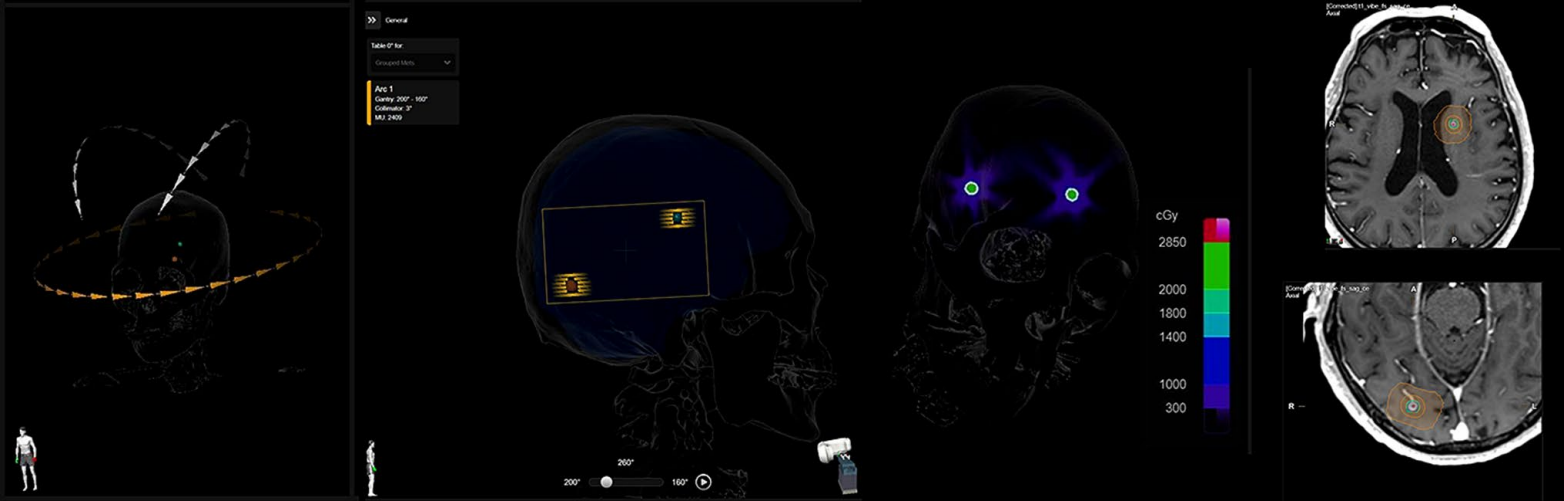
Single isocenter stereotactic irradiation using multiple brain mets SRS

The second approach is to use a treatment-planning device specialized for SI-STI-MBM with conformal arcs [33] (Fig. 2). The specialized treatment-planning system adopts an inverse-planning method and optimizes the collimator angles, couch angles, number of arcs, selection of the arc that irradiates the lesions, and the leaf motion for each target. Because of the utilization of dynamic conformal arc therapy for each lesion, leaf motion is not as complex as that of VMAT, and there is no need to conduct quality assurance (QA) for VMAT. As a result, SI-STI-MBM using dynamic conformal arcs can be performed quickly after computed tomography simulation, and the burden for medical physicists to conduct QA can be reduced. As this exclusive treatment-planning system of SI-STI-MBM with dynamic conformal arcs, Multiple Brain Mets SRS (BrainLab AG, Munich, Germany) has been released and is clinically used worldwide [34].

## Clinical outcomes of SI-STI-MBM

Several publications have examined the clinical outcomes of SI-STI-MBM for multiple brain metastases [26, 34–39]. Table 1 summarizes clinical outcomes of SI-STI-MBM. The local control rate at 12 months ranged from 82 to 97.5%, and toxicities above grade 2 occurred in 8% of patients. Although most reports were from retrospective studies, Kim et al. conducted a prospective study examining patients with 4–10 brain metastases [38]. They reported that the local recurrence rate at 12 months was 5% for all lesions, with no grade 3–5 treatment-related adverse events [38]. Therefore, SI-STI-MBM seems to achieve high local control with a low incidence of severe complications when compared with conventional LINAC-based stereotactic irradiation. Although the low-dose irradiated volume of the normal brain was larger in SI-STI-MBM than in GKS, target conformity and high-dose irradiated volume of the normal brain were similar to GKS [27, 28, 40]. The clinical effects of a larger low-dose irradiated volume when compared with that of GKS, remain unknown.

**Table 1:** Clinical outcomes of SI-STI-MBM with LINAC

Authors (publication year)	Number of patients	Number of metastases	Median dose	Overall survival	Local control	Toxicities
Nath et al. [26] (2010)	26	138 (range 2–13)	Median 18 Gy/1 fr (range 14–25 Gy/1–5 fr.)	6 m: 50% 12 m: 38%	6 m: 97% 12 m: 83%	Grade 3: 8%
Lau et al. [33] (2015)	15	Median 3 (range 2–13)	20 Gy/1 fr 3 cases: SRT	6 m: 60%	6 m: 92% 12 m: 82%	No grade 3 or 4
Serna et al. [34] (2015)	52	Total 87 (range 1–3)	12–20 Gy/1 fr	Median 7.2 m	82%	NA
Palmer et al. [37] (2020)	173	1014 (median 3, range 3–20)	18–24 Gy/1 fr 21–27 Gy/3fr 25–30 Gy/5fr	Median 13.4 m	12 m 99%	Grade 2: 1.4% sGrade 3: 0.9%
Kraft et al. [35] (2021)	140	Total 708	SRS 18–20 Gy SRT 30 Gy/5 fr	Median 15.8 m	12 m: 94%	NA
Bodensohn et al. [32] (2021)	65	254 (range 2–12)	SRS 15–20 Gy	Median 15 m	12 m: 97.5%	Grade 2: 6.2%, Grade 3: 4.6%
Kim et al. [36] (2021)	40	252 (range 4–10)	SRS: 22.7 Gy/1 fr. (24 pt.) SRT: 29.0 Gy/5 fr. (16 pt.)	Median 8.5 m	12 m: 95%	No grade 3–5

*SI-STI-MBM* single isocenter stereotactic irradiation for multiple brain metastases, *LINAC* linear accelerators, *fr.* fractions, *SRT* stereotactic radiotherapy, *SRS* stereotactic radiosurgery, *NA* not available, *pt.* patients

While only brain metastases are usually irradiated in SI-STI-MBM, a new approach that combines SI-STI-MBM and WBRT using a simultaneous integrated boost (SIB) method is applied in some institutions [41, 42]. In a phase 2 trial conducted in Canada, 47.5 Gy in 5 fractions was delivered to each brain metastasis, and the whole brain was covered with 20 Gy in 5 fractions simultaneously using the SIB method [42]. The local control rate at 12 months was 88%, and the severe radionecrosis above grade 2 was only 1.9% in nondeep lesions. Although these outcomes appeared tolerable, grade 3–5 radionecrosis occurred in 25% of patients with deep brain metastases, including basal ganglia and thalamus. In this study, the planning target volume (PTV) was created by adding 2 mm margin to the gross tumor volume (GTV) for brain and brainstem metastases. When brain metastases are located in eloquent or deep areas, radionecrosis and local recurrence directly affect neurological symptoms, and it is better to pay more attention to delineate contours and add margins to create PTV. Zhong et al. reported the clinical outcomes and quality of life of patients with brain metastases who underwent WBRT with SIB boost [41]. Although only 13 patients were included, the local control rate per lesion at 12 months was 98.6% and no adverse events above grade 2 were observed. In addition, there was no significant cognitive decline among the included patients during the median follow-up period of 11 months. Hippocampal-avoidance WBRT (HA-WBRT) can be delivered to patients with brain metastases, and a clinical trial investigating the benefits of delivering higher doses to brain metastases in HA-VMAT using the SIB method is ongoing in Singapore [43]. Although it is unclear whether WBRT combined with SI-STI-MBM is suitable, the advancement of radiation oncology may offer more options to treat brain metastases in a less invasive manner.

## Specific consideration points in SI-STI-MBM; offset and small fields

There are two major physics issues in SI-STI-MBM.

First, all lesions or all except one target must be offset in SI-STI-MBM. Translational and rotational errors in offset lesions result in larger positional displacement and dose coverage impairment as the distance between the isocenter and center of the target increases [44–46]. Therefore, correction of these errors should be considered in offset lesions in SI-STI-MBM. To minimize these errors, 6D positioning correction using ExacTrac X-ray system after every couch rotation can contribute to highly accurate positioning within a short time. Tsuruta et al. reported that there was no significant difference in GTV D_99.5%_ and D_0.5%_ despite the distance between the target and the isocenter in SI-STI-MBM with VMAT when 6D positioning correction was performed using the ExacTrac X-ray system [47]. With regard to adding a margin to the GTV to compensate for these errors, 1 mm seems to be sufficient from the point of view of physical aspects [46, 48]. Kraft et al. reported that clinical outcomes of SI-STI-MBM using 6D positioning correction with a 1 mm margin added to create PTV [37]. In their report, the local control rate at 12 months was 94%, and the distance between the isocenter and tumors had little effect on local control. These data imply that the 6D positioning correction and a 1 mm margin added to PTV made the translational and rotational errors clinically acceptable. Based on these reports, there is little need to give a larger margin in SI-STI-MBM when compared with conventional STI, as long as proper setup and position correction are performed.

Second, the inaccuracy of small fields generated by the optimization of SI-STI-MBM with VMAT should be considered. The leaf motion in SI-STI-MBM with VMAT is complex, and many small fields are generated for multiple targets. Research on small fields of stereotactic irradiation is ongoing, and information including ICUR 91 supports the QA of stereotactic irradiation for small tumors [49, 50]. Although much effort has been made to establish QA in SI-STI-MBM with VMAT, the uncertainty in small-field irradiation cannot be ignored. Therefore, clinical outcomes should be analyzed and confirmed in short- and long-term follow-ups at each institution.

## Prospects in SI-STI-MBM

Additional evidence is required to evaluate the usefulness of stereotactic irradiation, including SI-STI-MBM. Here, we addressed the prospects of stereotactic irradiation, not limited to SI-STI-MBM, but also including conventional LINAC-based stereotactic irradiation and GKS for multiple brain metastases.

First, there is currently limited high-level evidence that directly compares WBRT and stereotactic irradiation in patients with 5–10 brain metastases. Although JLGK0901 showed the usefulness of stereotactic irradiation in patients with 5–10 brain metastases, there were no randomized controlled trials (RCTs) comparing WBRT versus stereotactic irradiation for more than three metastases, before the publication of the Dutch phase III RCT reported by Hartgerink et al. [51]. In a Dutch Phase III RCT, WBRT and stereotactic irradiation were compared in patients with 4–10 brain metastases, and the primary endpoint was quality of life (QOL) 3 months after completion of radiotherapy. Although the planned sample size was 230 (115 patients per group), patient recruitment was poor, and the study included 29 patients. The primary endpoint was not met, and there was no difference between the two groups, while the statistical power was weak due to poor accrual. The preference of radiation oncologists, patients, and referrers negatively affected patient recruitment in this clinical trial. Therefore, it may be challenging to conduct RCTs comparing WBRT and stereotactic irradiation for limited brain metastases. However, two RCTs are currently underway to investigate the feasibility and merits of stereotactic irradiation over WBRT in patients with more than four brain metastases. One RCT was the CE.7 study conducted by the Canadian Cancer Trials Group, which compared stereotactic irradiation with hippocampus-avoidance WBRT at 30 Gy in 10 fractions (ClinicalTrials.gov number NCT03550391). The number of brain metastases ranged from five to 15, and stereotactic irradiation was performed in a single fraction. The primary endpoint is overall survival (OS). Another clinical trial, which also compared stereotactic irradiation and WBRT, is now being conducted at the Dana-Farber Cancer Institute and Brigham and Women’s Hospital, and patients with 5–20 brain metastases are registered in this trial (ClinicalTrials.gov number NCT03075072). The primary endpoint was QOL 6 months after the completion of radiotherapy.

Second, the adaptation of SI-STI-MBM for more than 10 brain metastases is unclear. From a technical perspective, SI-STI-MBM can be adapted for more than 10 brain metastases. However, the superiority or noninferiority of stereotactic irradiation over WBRT in patients with more than 10 brain metastases remains unknown. These two RCTs included patients with more than 10 brain metastases. If these two trials are completed with sufficient statistical power, the treatment strategy for more than 10 brain metastases may change.

Finally, brain metastases from small-cell lung carcinoma are controversial in terms of the benefits from stereotactic irradiation. This is because brain metastases from small-cell lung carcinomas tend to occur more frequently than those from nonsmall-cell lung carcinomas. Therefore, WBRT is the standard treatment for brain metastases from cell lung carcinoma. Recently, a multicenter retrospective cohort study named the FIRE-SCLC cohort study was published [52]. In this study, 710 patients treated with stereotactic irradiation without WBRT or prophylactic cranial irradiation from 1994 to 2018 were included. The median OS was 8.5 months, and it was similar between patients with 2–4 brain metastases and patients with 5–10 brain metastases, consistent with the JLGK0901 study [25]. Although the FIRE-SCLC cohort study is just a retrospective study, the clinical results of stereotactic irradiation seem clinically acceptable and suggest the feasibility of stereotactic irradiation as a reasonable treatment option for brain metastases from small-cell lung carcinoma. Moreover, two ongoing phase II studies investigate the feasibility of stereotactic irradiation in patients with brain metastases from small-cell lung carcinoma in Germany and the USA (ClinicalTrials.gov numbers NCT03297788 and NCT03391362). These clinical cases include patients with 1–10 brain metastases. In addition, Gondi et al. conducted a phase III trial comparing hippocampal-avoidance WBRT and stereotactic irradiation for 10 or fewer brain metastases from small-cell lung cancer (NRC CC009, ClinicalTrials.gov number NCT04804644). If the benefit of stereotactic irradiation is shown from these clinical trials, not only conventional stereotactic irradiation but also SI-STI-MBM can be a useful treatment in patients with multiple brain metastases from small-cell lung cancer.

## Conclusion

As the prognosis of patients with brain metastases improves, stereotactic irradiation, rather than WBRT, has been performed in many patients to achieve long-term local control and avoid neurocognitive dysfunction due to radiotherapy. Recent advances in radiotherapy devices, treatment-planning systems, and image-guided radiotherapy have enabled SI-STI-MBM to deliver ablative high doses to multiple brain metastases within a short treatment time. In SI-STI-MBM, local control is excellent and the rate of adverse events, including radionecrosis, is tolerable. As SI-STI-MBM uses only one isocenter and many targets are offset, clinicians, physicists, and radiotherapists should reduce translational and rotational errors as much as possible by using a highly precise image-guided technique, including 6D positional correction. High-level evidence regarding stereotactic irradiation for more than 11 brain metastases and brain metastases from small-cell lung cancers will be shown in ongoing clinical trials.

## References

[1] Barnholtz-Sloan JS, Sloan AE, Davis FG, Vigneau FD, Lai P, Sawaya RE (2004). Incidence proportions of brain metastases in patients diagnosed (1973 to 2001) in the metropolitan detroit cancer surveillance system. J Clin Oncol.

[2] Tsao MN, Lloyd NS, Wong RK (2005). Supportive care guidelines group of cancer care Ontario’s program in evidence based C. clinical practice guideline on the optimal radiotherapeutic management of brain metastases. BMC Cancer.

[3] Zhong X, Huang B, Feng J, Yang W, Liu H (2015). Delayed leukoencephalopathy of non-small cell lung cancer patients with brain metastases underwent whole brain radiation therapy. J Neurooncol.

[4] Sperduto PW, Yang TJ, Beal K, Pan H, Brown PD, Bangdiwala A (2017). Estimating survival in patients with lung cancer and brain metastases: an update of the graded prognostic assessment for lung cancer using molecular markers (lung-molGPA). JAMA Oncol.

[5] Leksell L (1983). Stereotactic radiosurgery. J Neurol Neurosurg Psychiatry.

[6] Ogura K, Mizowaki T, Ogura M, Sakanaka K, Arakawa Y, Miyamoto S (2012). Outcomes of hypofractionated stereotactic radiotherapy for metastatic brain tumors with high risk factors. J Neurooncol.

[7] Semwal MK, Singh S, Sarin A, Bhatnagar S, Pathak HC (2012). Comparative clinical dosimetry with X-knife and gamma knife. Phys Med.

[8] Torizuka D, Uto M, Takehana K, Mizowaki T (2021). Dosimetric comparison among dynamic conformal arc therapy, coplanar and non-coplanar volumetric modulated arc therapy for single brain metastasis. J Radiat Res.

[9] Clark GM, Popple RA, Young PE, Fiveash JB (2010). Feasibility of single-isocenter volumetric modulated arc radiosurgery for treatment of multiple brain metastases. Int J Radiat Oncol Biol Phys.

[10] Gaspar L, Scott C, Rotman M, Asbell S, Phillips T, Wasserman T (1997). Recursive partitioning analysis (RPA) of prognostic factors in three radiation therapy oncology group (RTOG) brain metastases trials. Int J Radiat Oncol Biol Phys.

[11] Sperduto PW, Berkey B, Gaspar LE, Mehta M, Curran W (2008). A new prognostic index and comparison to three other indices for patients with brain metastases: an analysis of 1960 patients in the RTOG database. Int J Radiat Oncol Biol Phys.

[12] Sperduto PW, Kased N, Roberge D, Xu Z, Shanley R, Luo X (2012). Summary report on the graded prognostic assessment: an accurate and facile diagnosis-specific tool to estimate survival for patients with brain metastases. J Clin Oncol.

[13] Sperduto PW, De B, Li J, Carpenter D, Kirkpatrick J, Milligan M (2022). Graded prognostic assessment (GPA) for patients with lung cancer and brain metastases: initial report of the small cell lung cancer GPA and update of the non-small cell lung cancer GPA including the effect of programmed death ligand 1 and other prognostic factors. Int J Radiat Oncol Biol Phys.

[14] Sperduto PW, Jiang W, Brown PD, Braunstein S, Sneed P, Wattson DA (2017). Estimating survival in melanoma patients with brain metastases: an update of the graded prognostic assessment for melanoma using molecular markers (melanoma-molGPA). Int J Radiat Oncol Biol Phys.

[15] Sperduto PW, Fang P, Li J, Breen W, Brown PD, Cagney D (2019). Estimating survival in patients with gastrointestinal cancers and brain metastases: an update of the graded prognostic assessment for gastrointestinal cancers (GI-GPA). Clin Transl Radiat Oncol.

[16] Sperduto PW, Mesko S, Li J, Cagney D, Aizer A, Lin NU (2020). Beyond an updated graded prognostic assessment (breast GPA): a prognostic index and trends in treatment and survival in breast cancer brain metastases from 1985 to today. Int J Radiat Oncol Biol Phys.

[17] Khuntia D, Brown P, Li J, Mehta MP (2006). Whole-brain radiotherapy in the management of brain metastasis. J Clin Oncol.

[18] Brown PD, Jaeckle K, Ballman KV, Farace E, Cerhan JH, Anderson SK (2016). Effect of radiosurgery alone vs radiosurgery with whole brain radiation therapy on cognitive function in patients with 1 to 3 brain metastases: a randomized clinical trial. JAMA.

[19] Brown PD, Ballman KV, Cerhan JH, Anderson SK, Carrero XW, Whitton AC (2017). Postoperative stereotactic radiosurgery compared with whole brain radiotherapy for resected metastatic brain disease (NCCTG N107C/CEC·3): a multicentre, randomised, controlled, phase 3 trial. Lancet Oncol.

[20] Churilla TM, Ballman KV, Brown PD, Twohy EL, Jaeckle K, Farace E (2017). stereotactic radiosurgery with or without whole-brain radiation therapy for limited brain metastases: a secondary analysis of the north central cancer treatment group n0574 (alliance) randomized controlled trial. Int J Radiat Oncol Biol Phys.

[21] Aoyama H, Shirato H, Tago M, Nakagawa K, Toyoda T, Hatano K (2006). Stereotactic radiosurgery plus whole-brain radiation therapy vs stereotactic radiosurgery alone for treatment of brain metastases: a randomized controlled trial. JAMA.

[22] Kocher M, Soffietti R, Abacioglu U, Villa S, Fauchon F, Baumert BG (2011). Adjuvant whole-brain radiotherapy versus observation after radiosurgery or surgical resection of one to three cerebral metastases: results of the EORTC 22952–26001 study. J Clin Oncol.

[23] Andrews DW, Scott CB, Sperduto PW, Flanders AE, Gaspar LE, Schell MC (2004). Whole brain radiation therapy with or without stereotactic radiosurgery boost for patients with one to three brain metastases: phase III results of the RTOG 9508 randomised trial. Lancet.

[24] Chang WS, Kim HY, Chang JW, Park YG, Chang JH (2010). Analysis of radiosurgical results in patients with brain metastases according to the number of brain lesions: is stereotactic radiosurgery effective for multiple brain metastases?. J Neurosurg.

[25] Yamamoto M, Serizawa T, Shuto T, Akabane A, Higuchi Y, Kawagishi J (2014). Stereotactic radiosurgery for patients with multiple brain metastases (JLGK0901): a multi-institutional prospective observational study. Lancet Oncol.

[26] Nath SK, Lawson JD, Simpson DR, Vanderspek L, Wang JZ, Alksne JF (2010). Single-isocenter frameless intensity-modulated stereotactic radiosurgery for simultaneous treatment of multiple brain metastases: clinical experience. Int J Radiat Oncol Biol Phys.

[27] Thomas EM, Popple RA, Wu X, Clark GM, Markert JM, Guthrie BL (2014). Comparison of plan quality and delivery time between volumetric arc therapy (rapidarc) and gamma knife radiosurgery for multiple cranial metastases. Neurosurgery.

[28] Liu H, Andrews DW, Evans JJ, Werner-Wasik M, Yu Y, Dicker AP (2016). Plan quality and treatment efficiency for radiosurgery to multiple brain metastases: non-coplanar rapidArc vs gamma knife. Front Oncol.

[29] Ruggieri R, Naccarato S, Mazzola R, Ricchetti F, Corradini S, Fiorentino A (2018). Linac-based VMAT radiosurgery for multiple brain lesions: comparison between a conventional multi-isocenter approach and a new dedicated mono-isocenter technique. Radiat Oncol.

[30] Ballangrud A, Kuo LC, Happersett L, Lim SB, Beal K, Yamada Y (2018). Institutional experience with SRS VMAT planning for multiple cranial metastases. J Appl Clin Med Phys.

[31] Kishi N, Nakamura M, Hirashima H, Mukumoto N, Takehana K, Uto M (2020). Validation of the clinical applicability of knowledge-based planning models in single-isocenter volumetric-modulated arc therapy for multiple brain metastases. J Appl Clin Med Phys.

[32] Ziemer BP, Sanghvi P, Hattangadi-Gluth J, Moore KL (2017). Heuristic knowledge-based planning for single-isocenter stereotactic radiosurgery to multiple brain metastases. Med Phys.

[33] Huang Y, Chin K, Robbins JR, Kim J, Li H, Amro H (2014). Radiosurgery of multiple brain metastases with single-isocenter dynamic conformal arcs (SIDCA). Radiother Oncol.

[34] Bodensohn R, Kaempfel AL, Fleischmann DF, Hadi I, Hofmaier J, Garny S (2021). Simultaneous stereotactic radiosurgery of multiple brain metastases using single-isocenter dynamic conformal arc therapy: a prospective monocentric registry trial. Strahlenther Onkol.

[35] Lau SK, Zakeri K, Zhao X, Carmona R, Knipprath E, Simpson DR (2015). Single-isocenter frameless volumetric modulated arc radiosurgery for multiple intracranial metastases. Neurosurgery.

[36] Serna A, Escolar PP, Puchades V, Mata F, Ramos D, Gomez MA (2015). Single fraction volumetric modulated arc radiosurgery of brain metastases. Clin Transl Oncol.

[37] Kraft J, van Timmeren JE, Mayinger M, Frei S, Borsky K, Stark LS (2021). Distance to isocenter is not associated with an increased risk for local failure in LINAC-based single-isocenter SRS or SRT for multiple brain metastases. Radiother Oncol.

[38] Kim GJ, Buckley ED, Herndon JE, Allen KJ, Dale TS, Adamson JD (2021). Outcomes in patients with 4 to 10 brain metastases treated with dose-adapted single-isocenter multitarget stereotactic radiosurgery: a prospective study. Adv Radiat Oncol.

[39] Palmer JD, Sebastian NT, Chu J, DiCostanzo D, Bell EH, Grecula J (2020). Single-isocenter multitarget stereotactic radiosurgery is safe and effective in the treatment of multiple brain metastases. Adv Radiat Oncol.

[40] McDonald D, Schuler J, Takacs I, Peng J, Jenrette J, Vanek K (2014). Comparison of radiation dose spillage from the gamma knife perfexion with that from volumetric modulated arc radiosurgery during treatment of multiple brain metastases in a single fraction. J Neurosurg.

[41] Zhong J, Waldman AD, Kandula S, Eaton BR, Prabhu RS, Huff SB (2020). Outcomes of whole-brain radiation with simultaneous in-field boost (SIB) for the treatment of brain metastases. J Neurooncol.

[42] Nichol A, Ma R, Hsu F, Gondara L, Carolan H, Olson R (2016). Volumetric radiosurgery for 1 to 10 brain metastases: a multicenter, single-arm, phase 2 study. Int J Radiat Oncol Biol Phys.

[43] Chia BSH, Leong JY, Ong ALK, Lim C, Poon SH, Chua MLK (2020). Randomised prospective phase II trial in multiple brain metastases comparing outcomes between hippocampal avoidance whole brain radiotherapy with or without simultaneous integrated boost: HA-SIB-WBRT study protocol. BMC Cancer.

[44] Roper J, Chanyavanich V, Betzel G, Switchenko J, Dhabaan A (2015). Single-isocenter multiple-target stereotactic radiosurgery: risk of compromised coverage. Int J Radiat Oncol Biol Phys.

[45] Chang J, Wernicke AG, Pannullo SC (2018). Restricted single isocenter for multiple targets dynamic conformal arc (RSIMT DCA) technique for brain stereotactic radiosurgery (SRS) planning. J radiosurg SBRT.

[46] Chang J (2017). A statistical model for analyzing the rotational error of single isocenter for multiple targets technique. Med Phys.

[47] Tsuruta Y, Nakamura M, Nakata M, Hirashima H, Zhou D, Uto M (2022). Evaluation of correlation between intrafractional residual setup errors and accumulation of delivered dose distributions in single isocenter volumetric modulated arc therapy for multiple brain metastases. Phys Med.

[48] Guckenberger M, Roesch J, Baier K, Sweeney RA, Flentje M (2012). Dosimetric consequences of translational and rotational errors in frame-less image-guided radiosurgery. Radiat Oncol.

[49] Culcasi R, Baran G, Dominello M, Burmeister J (2021). Stereotactic radiosurgery commissioning and QA test cases-A TG-119 approach for Stereotactic radiosurgery. Med Phys.

[50] Wilke L, Andratschke N, Blanck O, Brunner TB, Combs SE, Grosu AL (2019). ICRU report 91 on prescribing, recording, and reporting of stereotactic treatments with small photon beams : statement from the DEGRO/DGMP working group stereotactic radiotherapy and radiosurgery. Strahlenther Onkol.

[51] Hartgerink D, Bruynzeel A, Eekers D, Swinnen A, Hurkmans C, Wiggenraad R (2021). A Dutch phase III randomized multicenter trial: whole brain radiotherapy versus stereotactic radiotherapy for 4–10 brain metastases. Neurooncol Adv.

[52] Rusthoven CG, Yamamoto M, Bernhardt D, Smith DE, Gao D, Serizawa T (2020). Evaluation of first-line radiosurgery vs whole-brain radiotherapy for small cell lung cancer brain metastases: the FIRE-SCLC cohort study. JAMA Oncol.

